# Development of Japanese utility weights for the Adult Social Care Outcomes Toolkit (ASCOT) SCT4

**DOI:** 10.1007/s11136-019-02287-6

**Published:** 2019-09-04

**Authors:** Takeru Shiroiwa, Yoko Moriyama, Hiromi Nakamura-Thomas, Mie Morikawa, Takashi Fukuda, Laurie Batchelder, Eirini-Christina Saloniki, Juliette Malley

**Affiliations:** 1 Center for Outcomes Research and Economic Evaluation for Health (C2H), National Institute of Public Health, 2-3-6 Minami, Wako, Saitama 351-0197 Japan; 2 Department of Health and Welfare Services, National Institute of Public Health, 2-3-6 Minami, Wako, Saitama 351-0197 Japan; 3 Graduate School of Health, Medicine and Welfare, School of Occupational Therapy, Saitama Prefectural University, 820 Sannomiya, Koshigaya, Saitama 343-8540 Japan; 4 Department of Policy Studies, Tsuda University, 1-18-24 Sendagaya, Shibuya-ku, Tokyo, 151-0051 Japan; 5 Personal Social Services Research Unit (PSSRU), Cornwallis Central, University of Kent, Canterbury, Kent CT2 7NF UK; 6 Personal Social Services Research Unit (PSSRU), London School of Economics and Political Science (LSE), Houghton Street, London, WC2A 2AE UK

**Keywords:** ASCOT, Preference, Best–worst scaling (BWS), Time trade-off (TTO), Quality of life, Social care, Social care-related quality of life (SCRQoL)

## Abstract

**Purpose:**

In developed countries, progressive rapid aging is increasing the need for social care. This study aimed to determine Japanese utility weights for the Adult Social Care Outcomes Toolkit (ASCOT) four-level self-completion questionnaire (SCT4).

**Methods:**

We recruited 1050 Japanese respondents from the general population, stratified by sex and age, from five major cities. In the best–worst scaling (BWS) phase, respondents ranked various social care-related quality of life (SCRQoL) states as “best,” “worst,” “second-best,” or “second-worst,” as per the ASCOT. Then, respondents were asked to evaluate eight different SCRQOL states by composite time-trade off (cTTO). A mixed logit model was used to analyze BWS data. The association between cTTO and latent BWS scores was used to estimate a scoring formula that would convert BWS scores to SC-QALY (social care quality-adjusted life year) scores.

**Results:**

Japanese BWS weightings for ASCOT-SCT4 were successfully estimated and found generally consistent with the UK utility weights. However, coefficients on level 3 of “Control over daily life” and “Occupation” domains differed markedly between Japan and the UK. The worst Japanese SCRQoL state was lower than that for the UK, as Japanese cTTO results showed more negative valuations. In general, Japanese SC-QALY score (for more than 90% of health states) was lower than that for the UK.

**Conclusions:**

We successfully obtained Japanese utility weights for ASCOT SCT4. This will contribute to the measurement and understanding of social care outcomes.

**Electronic supplementary material:**

The online version of this article (10.1007/s11136-019-02287-6) contains supplementary material, which is available to authorized users.

## Introduction

Many developed countries are facing rapid population aging, with Japan being one of the world’s fastest-aging countries. The proportion of elderly people (aged 65 and above) in 2016 was 27.3% in Japan [1], 23% in Italy, and 21% in Germany, Portugal, and Finland [2]. Meanwhile, with a negative population growth rate, the proportion of elderly people in Japan is expected to increase even more in the next 50 years, reaching an estimated 38.4% in 2065. In addition, a quarter of the entire population will be aged 75 years and above (these people are defined as “elderly in the latter stage of life” in Japan) [3]. The Japanese government updated its projected future social security costs in May 2018, and found that long-term care costs will increase to 2.5 times the 2018 costs by 2040 (from 1.9 to 3.3% of GDP). Thus, rapid aging will give rise to issues pertaining to financial and social sustainability in Japan. This situation is not limited to Japan; given the speed of population aging, other countries, including Asian countries such as China, South Korea, and Thailand, will face similar issues in the future.

In Japan, outcomes of long-term care are often evaluated based on activities of daily living (ADLs) including the Barthel Index [4] or instrumental activities of daily living (IADLs), which are measured by experts, not care service users. However, user-reported quality of life (QoL), an important maintenance or improvement goal for social care programs, is rarely assessed. While many measures have been developed for health-related QoL (HRQoL) [5–9], no standardized measure for measuring QoL of care service users currently exists. Recently, a research group at the University of Kent, in the United Kingdom, developed the Adult Social Care Outcomes Toolkit (ASCOT) [10–12], which is designed to measure social care–related QoL (SCRQoL). We developed a Japanese version of the ASCOT four-level self-completion questionnaire (SCT4) in 2017, with subsequent linguistic validation [9]. The development of a Dutch version has also been reported [13].

The ASCOT is a preference-based measure; that is, responses from care users can be converted to QoL scores (we sometimes call ASCOT scores “SC-QALY” or “social care quality-adjusted life years” scores) based on multi-attribute utility theory (MAUT) [14]. Based on quality-adjusted life year (QALY) calculations, obtained scores can be applied to economic evaluations pertaining to care programs. Economic evaluation of social care programs is not frequently performed in Japan, although it is important to evaluate the efficiency of long-term care programs under circumstances of rapidly increasing social care costs. Utility weights for ASCOT have been developed for the UK [10], but not for Japan. As utility weights might differ between countries due to differences in population characteristics and potential issues with questionnaire translation [15], it is important to develop utility weights tailored to each country, and compare them across countries to better understand differences in preferences for ASCOT states among countries or regions. To this end, we conducted a study to determine Japanese utility weights for ASCOT-SCT4.

## Methods

### ASCOT-SCT4

The Japanese version of the ASCOT-SCT4 was used in the study, with permission from and in collaboration with the developer of the original measure, the ASCOT team at the Personal Social Services Research Unit (PSSRU) at the University of Kent, and the copyright holder, the University of Kent. The Japanese version of ASCOT was completed using the following process. First, we translated the UK version of ASCOT SCT4 into Japanese and had it back-translated it into English by a different translator. Then, based on discussion among the developer, the Japanese research team, and the translation company, we produced a pre-final version, which was used for cognitive debriefing through interviewing a small number of potential users of ASCOT. We confirmed the tool’s linguistic validity and finalized the Japanese version after adjustment from a clinical perspective.

The ASCOT-SCT4 (the Japanese version is provided in Appendix 1 in Electronic Supplementary Material) consists of eight domains, covering the following aspects of SCRQoL: control over daily life, personal cleanliness and comfort, food and drink, personal safety, social participation and involvement, occupation, accommodation cleanliness and comfort, and dignity. Each domain is represented by one item, except the dignity domain, which has two items. All items have four response options. Description of each level depends on each item; however, generally, the following descriptions are used: “can do (or is) as I want to do (or be),” “can do adequately (or is adequate),” “cannot adequately do (or is not adequate),” “cannot do (or isn’t) at all.”

The first dignity item (the 8th item overall, “how the user feels about the fact that they need social care”) is not used in the scoring of the ASCOT instrument [16]. During development of ASCOT, it was found that some respondents were using the 9th item (“how the user feels about the way they receive social care”) to express that they did not like needing help with aspects of life. The 8th item thus has a role of not only allowing respondents to express unhappiness with needing help but also helping them to answer the 9th item in the way that the tool intended.

### Best–worst scaling and time trade-off

We measured preferences among ASCOT states in the general population by best–worst scaling (BWS) and composite time trade-off (cTTO), as described by Netten et al. [10]. BWS [17, 18] has been increasingly used to construct utility weights [19, 20]. According to a systematic review [21], 62 BWS studies had been conducted in healthcare research as of April 2016. In the area of social care, preference patterns and utility weights based on BWS (explained in detail in the next paragraph) and discrete choice experiment (DCE) (in which respondents are asked to choose one profile combining each domain between two shown profiles) are reportedly similar [22]. However, in this case, a problem with DCE was that the number of domains was too large to compare two profiles. As cognitive burden seems higher for respondents to the 8-domain DCE than to BWS, it was less feasible for the pre-survey. In addition, BWS can obtain more preference information than DCE per task [17]. Therefore, we applied BWS to the ASCOT valuation survey.

**Fig. 1:**
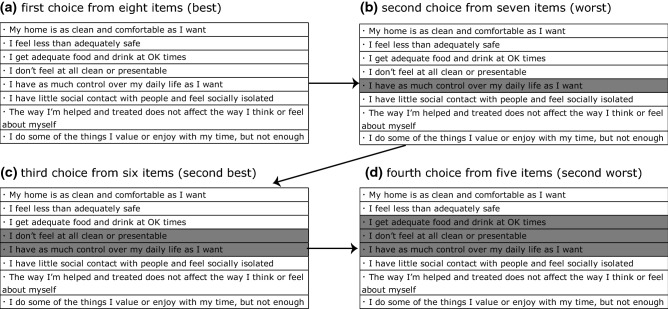
Example of BWS profile

In the BWS survey, respondents are asked to choose “best” and “worst” options among presented alternatives. That is, BWS methods are classified into the following three types: case 1 (object case), case 2 (profile case), and case 3 (multi-profile case) [18]. In this study, we used the case 2 method, showing a profile combining each ASCOT SCT4 domain (Fig. 1). This is because, on the one hand, preference for each item needs to be evaluated, whole on the other hand it is too complicated for respondents to compare profiles with too many domains. Respondents were asked to put themselves into an imaginary state (which is described by the profile) of being in need of care services, and then to select the best, worst, second-best, and second-worst domains in a sequential manner. That is, they first rated eight domains as “best,” and then the remaining seven, six, and five domains as “worst,” “second-best,” and “second-worst” respectively. Selected domains were grayed-out and the remaining domains were presented for the next choice.

The TTO method is used to measure health state preferences [23]. First, respondents are asked to imagine living with a particular health state (profiles combining eight ASCOT items in this survey) for a certain amount of time (e.g., 10 years) (life A). After that, respondents have to indicate the number of years living in full health (life B) at which the respondents feel indifferent between life A and life B. We selected TTO rather than standard gamble (SG) based on evidence about feasibility for respondents [24].

In this survey, respondents were asked to compare 10 years’ life with presented SCRQoL state (combining each ASCOT-SCT4 domain) with *x* years’ life with full SCRQoL state. We ascertained indifference between “x years’ life in full SCRQoL state” and “10 years’ life in the presented SCRQoL state,” increasing or decreasing the number of years. In the case of cTTO, the “worse than-dead” SCRQoL state was evaluated differently from the “better than-dead” state. To evaluate the former, cTTO employs lead-time TTO [25–27], which limits the minimum TTO score to − 1. When respondents were asked about the period of life covered by a SCRQoL state, their responses were facilitated by a visual representation of the question (a bar graduated from 0 to 10, on which the respondents marked a particular number of years).

In the BWS phase, four blocks consisting of eight SCRQoL profiles (e.g., “24313222” is a profile with second level of control, forth (worst) level of personal care, etc.) were randomly allocated to each respondent [10]; 32 profiles were selected from all 4^8^ profiles using a fractional–factorial design (details should be referred to Netten et al. [10]) In the cTTO phase, eight blocks consisting of eight SCRQoL profiles (total 64 actually possible SCRQoL profiles) were similarly allocated to each respondent.

### Subjects and survey process

The computer-based survey was conducted in five representative, geographically dispersed major cities in Japan (Sapporo, Tokyo, Nagoya, Osaka, and Fukuoka) and targeted the general population. Respondents (aged 20 to 79) were recruited by a research company (Anterio, Inc.) based on non-random sampling of 1050 respondents throughout Japan (i.e., roughly 200 respondents at each location). The sample number was not based on any rigid statistical consideration, but referred to a UK survey (part of the Measuring Outcomes for Public Service Users (MOPSU) project) that carried out power analysis to determine sample size. Respondents were stratified by sex and age group, and interviews were performed using a computer- (tablet-) based, one-on-one setting over intervals of 30 to 60 min at each local survey center.

First, self-assessment of the respondent’s own SCRQoL using ASCOT occurred, followed by BWS questions. The respondent was asked to value eight SCRQoL profiles by BWS and then other eight by cTTO. The order of BWS and TTO was not randomized, and BWS was performed before TTO tasks. After the valuation process was completed, experience with social care and demographic data were collected from respondents. The order of domain descriptions for BWS SCRQoL profiles was randomized to avoid positioning effects. Response time was recorded for BWS and cTTO processes.

All responses were automatically collected as electronic data. The survey was conducted from February to March 2018. Prior to administering the survey, all investigators received training for approximately half a day at each location. To ensure quality and consistency among investigators, the number of investigators was limited to roughly five at each location.

This study was approved by the ethics committee of the National Institute of Public Health, to which the corresponding author belongs (NIPH-IBRA #12176).

### Statistical analysis

A panel mixed logit model [28] was used for the analysis of BWS data considering sequential choice by each respondent (model #1). A mixed logit model can consider heterogeneity of coefficients by relaxing the assumption of independence of irrelevant alternatives (IIA), whereas the simple multinomial logit (MNL) model assumes that all the responses are independent. In BWS tasks, it might be more appropriate if each respondent’s best and second-best and worst and second-worst choices are separately regarded as clusters than if all choices (including both the best and worst choices) are considered together for each respondent, because there may be heterogeneity between two best and worst choices each. We also developed a mixed logit model treating each respondent’s best or worst choices as different clusters (model #2). In addition, the simple MNL model was applied for sensitivity analysis (model #3).

Both mixed logit models and the MNL model include 7 (domain level; domain 5 is reference term) + 3 × 8 (item level; level 4 is reference term) = 31 parameters to be estimated based on the collected data. Therefore, when choices are analyzed based on random utility theory, *U*_ij_ (the utility respondent j derives from choosing item *i*) is divided into an explainable component (*V*_*ij*_) and a random component (*ε*_*ij*_).

$$ \begin{aligned} U_{ij} & = V_{ij} + \varepsilon_{ij} \\ V_{ij} & = \beta_{1} X_{1} + \beta_{2} X_{2} + \cdots + \beta_{8} X_{8} + \beta_{11} X_{11} + \beta_{12} X_{12} + \cdots + \beta_{83} X_{83} , \\ \end{aligned} $$

where *β*_*p*_ is common effects of the *p*th ASCOT domain (*p* = 1 accommodation, 2 safety, 3 food, 4 cleanliness, 6 participation, 7 dignity, 8 occupation) and *β*_*pq*_ is effects of the *q*th (1 ≤ *q* ≤ 3) level of the *p*th domain compared with the fourth level of the same domain. In the mixed logit model, let *β*_*p*_^m^ and *β*_*p*_^s^ be mean and scale parameters, respectively, for the random coefficient *β*_*p*_,

$$ \beta_{p} = \beta_{p}^{\text{m}} + \beta_{p}^{\text{s}} \cdot \eta , $$

where *η* is a stochastic compartment with normal distribution. We also performed using a log-normal distribution as model #4.

Although the number of items including a profile sequentially decreases from eight to five, data of four choices can be obtained from each respondent, i.e., we reflected the following situation in the coding: the best choice from among eight items, the worst choice from among seven items, the second best choice from among six items, and the second worst choice from among five items [10]. In case of the worst and second-worst choices, − 1 was used as *X*_*p*_ and *X*_*pq*_ if applicable [29]. Parameters in the utility function were estimated with *Mixedlogit* in STATA 15 using all the pooled choice data (best/worst and first/second). Respondents with a total BWS time of < 4.5 min, which was considered too short based on the pre-test results of a valuation survey in the UK, were excluded under the assumption that normal respondents could not complete eight BWS tasks (4 choices were needed per BWS task) within that time.

Next, we calculated *coefficients* of all 4 × 8 = 32 items. The coefficients of each item can be calculated by sum of domain and level effects. For example, in the case of level 2 in “personal cleanliness and comfort” (domain 2), coefficients are computed by the sum of coefficients of “domain 2” (*β*_2_) and “level 2 in domain 2” (*β*_22_). After that, coefficients of 32 items were rescaled to be first level of “Control over daily life” domain = 1 and fourth level of the same domain = 0, by linear transformation, which is similar to the method used for the UK. We call these transformed coefficients *weights*. Finally, we calculated *latent BWS scores* of cTTO 64 states using the sum of each domain’s applicable eight weights. Note that these latent BWS scores are not yet standardized to QoL scale (1 = full health and 0 = death); we need to convert them using cTTO data.

With regard to cTTO data, when respondents equated 10 years of life with better-than-dead SCRQoL state to *x* years of life with perfect health, the QoL score was calculated as *x*/10. Conversely, when *y* years of life with perfect SCRQoL was equated with “life with perfect SCRQoL for 10 years, followed by life with worse-than-dead SCRQoL state for 10 years,” then the QOL score was calculated as *y*/10 − 1. Mean QOL scores of 64 SCRQoL states were calculated; as in the BWS phase, respondents with a total cTTO time of < 5.0 min were excluded based on the pre-test results of a UK valuation survey.

Last, to convert the latent BWS scores to QOL scores (or SC-QALY scores), the linear relation function *f*(∙) between latent BWS scores and cTTO scores of 64 SCRQoL states was estimated; namely, TTO_*i*_=* f* (BWS_*i*_)+* ε*_*i*_, where TTO_*i*_ is the observed mean cTTO score, and BWS_*i*_ is the latent BWS score for the *i*th SCRQoL state (1 ≤ *i* ≤ 64). However, the linear function, *f*(*x*)=* ax *+* b*, has a restriction that the maximum ([11111111]) latent BWS score is converted to 1 (which is the definition of the maximum SC-QALY score).

## Results

The final sample included 1050 respondents adjusted for sex and age from five cities in Japan. Mean and median total response times to all the questions including BWS and TTO were 43.7 min (SD: 12.1) and 42 min (IQR 35–51 min), respectively; 83.6% of respondents completed all questions in 30 to 60 min.

Mean and median response times for all the BWS procedures were 10.6 min (SD: 4.7) and 9.6 min (IQR 7.3–12.7 min), respectively. Thirty-two (3.0%) respondents with total BWS times of < 4.5 min were excluded from the statistical analysis set. On the other hand, mean and median response times for the cTTO procedure were 10.7 min (SD: 44) and 10.1 min (IQR 7.6–13.3 min), respectively. Fifty-nine (5.6%) respondents with a total cTTO time of < 5.0 min were excluded from the statistical analysis set.

### Demographic factors

The background of the respondents was comparable with that of the general population. Table 1 summarizes respondent demographic factors. Household income of 49.3% of respondents was less than JPY 6 million (USD 55,000; USD 1 = JPY 110 as of July 2018) compared to a median household income of all Japanese families of JPY 5.4 million (USD 49,000) in 2016 [30]. The proportion of permanent full time workers was 39.8%, which is roughly comparable with 33.6% in the Japanese population, calculated by the “number of permanent full time workers/number of people aged 15 or over” according to government statistics in 2016 [31, 32]. The proportion of homemakers was 18.2%, which is similar with 13.5% in the Japanese population calculated by the “number of homemakers/number of people aged 15 or over” in 2016. Regarding education level, 43.6% of respondents had college, university, or graduate education, suggesting that approximately half of our respondents had received more than 14 years’ education (if vocational schools are also included, the rate is more than 50%). According to national statistics [33], the “number of people with college, university, or graduate education/number of people aged 20 years or over” in 2012 was 19.8%. Finally, 60.6% of our respondents were married, which is similar to 60.3% calculated by the “number of married people/number of people aged 20 or over” in 2015.

**Table 1:** Demographic characteristics of survey respondents

	Number	Percentage
Sex		
Male	525	50.0
Female	525	50.0
Age		
20–29	174	16.6
30–39	176	16.8
40–49	175	16.7
50–59	175	16.7
60–69	175	16.7
70–79	175	16.7
Region		
Tokyo	208	19.8
Sapporo	210	20.0
Fukuoka	211	20.1
Osaka	211	20.1
Nagoya	210	20.0
Employment		
Full-time worker (permanent)	397	37.8
Full-time worker (non-permanent)	53	5.0
Part-time worker	198	18.9
Self-employed or manager	63	6.0
Housemaker	191	18.2
Retired	95	9.0
Student	48	4.6
Other	5	0.5
Household income (JPY 10,000)		
< 100	25	2.4
100–200	48	4.6
200–400	210	20.0
400–600	234	22.3
600–1000	284	27.0
1000–1500	73	7.0
1500–2000	11	1.0
> 2000	6	0.6
No answer	159	15.1
Education		
Elementary or junior high school	35	3.3
High school	418	39.8
Vocational school	138	13.1
College	118	11.2
University	329	31.3
Graduate school	12	1.1
Marital status		
Unmarried	278	26.5
Married	636	60.6
Divorced/widowed	136	13.0

### BWS results

Table 2 shows the estimated coefficients of the BWS analysis in the both mixed logit model and MNL, in which the 4th (worst) level of “control over daily life” was used as the reference. Table 3 shows standardized Japanese BWS weightings generated from a linear transformation of estimated coefficients shown in Table 2; coefficients of levels 1 and 4 in “control over daily life” domain are 1 and 0, respectively. Table 3 derives from the coefficients from model #3 in Table 2, because the log likelihood of this model is the best and coefficients were not very different among the four models. The most preferred item was level 1 in the “occupation” domain, and the second-most preferred was level 1 in the same “control over daily life.” In UK, the most preferred item was level 1 in the “control over daily life” domain. In Japan, the least preferred item was level 4 in the “control over daily life” and “dignity” domains; the former item is the least preferred, similarly to the UK. All the coefficients in Table 2 were consistent; weights at the higher level in the same domain are higher, and that at the lower level, lower.

**Table 2:** Estimated coefficients from BWS responses

	Model #1		Model #2		Model #3		Model #4	
Statistical model	Multinomial logit		Mixed logit					
Cluster	-		Respondents		Respondents and choices			
Distribution	-		Normal				Log normal	
Parameter	Estimate	SE	Estimate	SE	Estimate	SE	Estimate	SE
Domain level								
Accommodation	0.1958	0.0485	0.2033	0.0492	0.2124	0.0572	− 0.4197	0.0736
Safety	0.2806	0.0496	0.2953	0.0533	0.3620	0.0585	− 0.3074	0.0699
Food	0.2492	0.0507	0.2845	0.0531	0.3487	0.0602	− 0.2085	0.0650
Cleanliness	0.4998	0.0500	0.5084	0.0508	0.5743	0.0566	− 0.0212	0.0640
Control	Ref.		Ref.		Ref.		Ref.	
Participation	0.1103	0.0491	0.1136	0.0501	0.1330	0.0553	− 0.4405	0.0750
Dignity	0.0954	0.0490	0.0914	0.0513	0.0005	0.0576	− 0.7710	0.1043
Occupation	0.3716	0.0509	0.4034	0.0529	0.5452	0.0582	− 0.0343	0.0534
Item level								
Accommodation								
Level 1	3.0961	0.0526	3.1756	0.0536	3.5226	0.0635	3.4131	0.0588
Level 2	2.7978	0.0531	2.8744	0.0540	3.1502	0.0634	3.0506	0.0595
Level 3	0.4620	0.0516	0.4708	0.0523	0.5459	0.0562	0.4031	0.0528
Safety								
Level 1	2.3374	0.0550	2.3536	0.0559	2.5613	0.0625	2.4852	0.0608
Level 2	1.0467	0.0543	1.0846	0.0547	1.2074	0.0587	1.0892	0.0558
Level 3	0.2143	0.0522	0.2390	0.0531	0.2753	0.0569	0.1670	0.0532
Food								
Level 1	3.0061	0.0541	3.0517	0.0552	3.2185	0.0645	3.0849	0.0607
Level 2	2.7677	0.0541	2.8139	0.0553	2.9394	0.0637	2.7967	0.0601
Level 3	0.5994	0.0532	0.6064	0.0540	0.6573	0.0572	0.4365	0.0536
Cleanliness								
Level 1	2.1248	0.0544	2.1886	0.0554	2.4154	0.0617	2.3738	0.0645
Level 2	1.9829	0.0553	2.0474	0.0563	2.2212	0.0623	2.1896	0.0652
Level 3	0.3392	0.0522	0.3483	0.0531	0.3894	0.0567	0.3370	0.0566
Control								
Level 1	3.6181	0.0534	3.7034	0.0543	4.0760	0.0586	4.6150	0.0541
Level 2	3.4475	0.0533	3.5336	0.0543	3.8895	0.0584	4.4245	0.0538
Level 3	0.3035	0.0512	0.3147	0.0519	0.3610	0.0545	0.9886	0.0467
Participation								
Level 1	2.8462	0.0537	2.9209	0.0546	3.1844	0.0615	3.0673	0.0589
Level 2	2.6323	0.0542	2.6982	0.0552	2.9607	0.0618	2.8377	0.0594
Level 3	1.1141	0.0537	1.1488	0.0545	1.2745	0.0579	1.1450	0.0556
Dignity								
Level 1	2.4354	0.0547	2.4768	0.0556	2.8675	0.0643	2.7330	0.0600
Level 2	1.1543	0.0533	1.1910	0.0541	1.4144	0.0592	1.2564	0.0557
Level 3	0.1962	0.0509	0.1998	0.0519	0.2387	0.0566	0.1264	0.0537
Occupation								
Level 1	3.3469	0.0543	3.4176	0.0555	3.6034	0.0641	3.5408	0.0614
Level 2	3.2118	0.0540	3.2695	0.0551	3.4306	0.0637	3.3692	0.0610
Level 3	0.3217	0.0531	0.3302	0.0540	0.3436	0.0564	0.2302	0.0543
Log likelihood	− 46,882		− 46,489		− 44,764		− 45,043	

*P* value of all the coefficients are less than 0.001

*SE* standard error

**Table 3:** Comparison of Japanese and UK BWS weightings

Item	Level	Japanese weight	UK weight
Control over daily life	1	1	1
	2	0.954	0.919
	3	0.089	0.541
	4	0	0
Personal cleanliness and comfort	1	0.734	0.911
	2	0.686	0.789
	3	0.236	0.265
	4	0.141	0.195
Food and drink	1	0.875	0.879
	2	0.807	0.775
	3	0.247	0.294
	4	0.086	0.184
Personal safety	1	0.717	0.880
	2	0.385	0.452
	3	0.156	0.298
	4	0.089	0.114
Social participation and involvement	1	0.814	0.873
	2	0.759	0.748
	3	0.345	0.497
	4	0.033	0.241
Occupation	1	1.018	0.962
	2	0.975	0.927
	3	0.218	0.567
	4	0.134	0.170
Accommodation cleanliness and comfort	1	0.916	0.863
	2	0.825	0.780
	3	0.186	0.374
	4	0.052	0.288
Dignity	1	0.704	0.847
	2	0.347	0.637
	3	0.059	0.295
	4	0.000	0.263

### cTTO results and conversion to SC-QALY scores

**Fig. 2:**
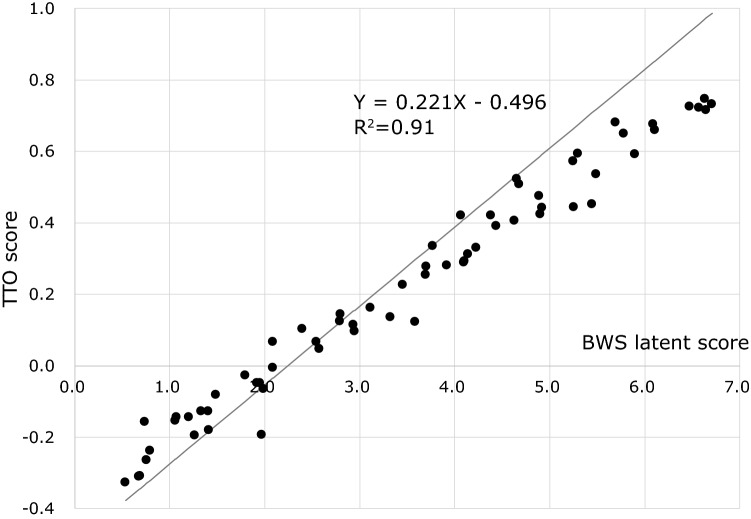
Relationship between latent BWS and TTO scores

Mean and median response times for the cTTO procedure were 10.7 min (SD: 44) and 10.1 min (IQR 7.6–13.3 min), respectively. Fifty-nine (5.6%) respondents with a total cTTO time of < 5.0 min were excluded from the statistical analysis. The worst TTO score was − 0.327 [44444444], and the best score was 0.746 [11121212]. Median of 64 TTO scores was 0.240. Twenty health states (31%) were evaluated as WTD; scores were less than 0. Figure 2 shows the relationships between latent BWS scores (calculated using Table 3) and cTTO scores of 64 states. Based on the linear relationship between latent BWS score and cTTO score, latent BWS scores can be converted to SC-QALY scores using the following formula:

$$  {\text{SC{-}QALY}}{\mkern 1mu} {\text{score}} = (0.221 \times {\text{latent}}{\mkern 1mu} {\text{BWS}}{\mkern 1mu} {\text{score}}{\mkern 1mu} {\text{calculated}}{\mkern 1mu} {\text{using}}{\mkern 1mu} {\text{Table}}{\mkern 1mu} 3) - 0.496.{\text{ }};{\text{Slope:}}\quad 0.221(t = 55.6,{\mkern 1mu} p;0.001),\quad {\text{intercept:}}\quad  - {\mkern 1mu} 0.496(t =  - 18.4,{\mkern 1mu} p;0.001),\quad R^{2}  = 0.91   $$

**Fig. 3:**
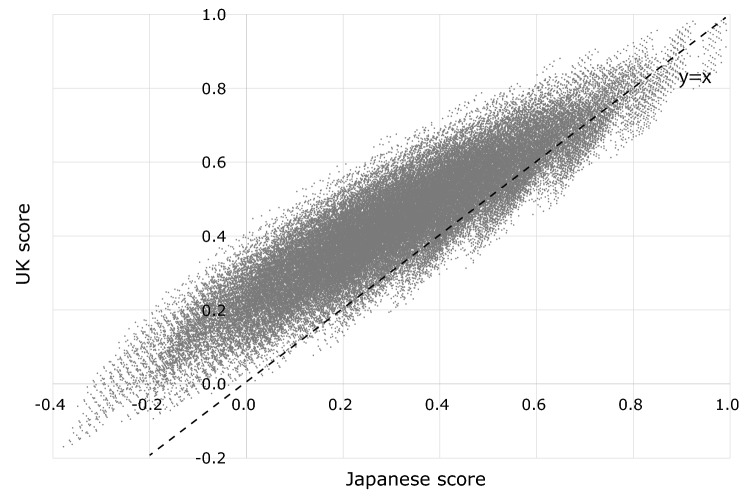
Comparison between Japanese and UK scores for all ASCOT patterns

SC-QALY scores were distributed between maximum and minimum SC-QALY scores of 1.00 and –0.38, respectively. Finally, we compared Japanese SC-QALY scores with UK scores for all 4^8^ = 65,536 patterns. Figure 3 shows a scatter plot of Japanese and UK scores; compared to Japanese SC-QALY scores, 91.0% of UK SC-QALY scores (*N* = 59,666) were higher. Pearson correlation coefficient and intraclass correlation coefficients (ICCs) were respectively 0.91 and 0.70 between Japanese and UK SC-QALY score. The calculation methods of ASCOT scores are illustrated in the “Appendix”.

## Discussion

In this study, we successfully determined Japanese utility weights for ASCOT SCT4 through a survey of 1050 participants who responded to both BWS and cTTO questions. These utility weights reflect Japanese preferences and can be used to calculate SC-QALY scores for economic evaluation. This means SCRQOL scores measured by ASCOT SCT4 can meet the requirements of Japanese official guidelines for the economic evaluation of drugs/medical devices (“the use of an instrument with a scoring algorithm developed in Japan is recommended”) [34].

The comparison of utility weights between Japan and the UK (Table 3) revealed consistency between Japanese and UK weightings. Pearson correlation coefficient was 0.91 and ICC was 0.70 between Japanese and UK scores. However, some coefficients greatly differed between Japan and the UK. For instance, the weight of level 3 in “control over daily life” and “occupation” domains greatly differed between Japan and the UK. This may reflect a Japanese preference called “pin pin korori,” which involves a wish for two things: “The first is a long, spry life. The second is a quick and painless death” (*The Economist*). The finding that Japanese weights for lower levels in the “dignity” domain tended to be smaller than UK weights lends further support to this interpretation. Generally, the third level’s weight in each domain (especially, occupation, accommodation, and dignity, in addition to control) was smaller in Japan than in the UK.

Conversion formulae for SC-QALY score, “SC-QALY score = (0.221 × latent BWS score) − 0.496” in Japan and “SC-QALY score = (0.203 × latent BWS score) − 0.466” in the UK, were also comparable. A comparison of SC-QALY scores revealed generally lower scores for Japanese compared to the UK. These lower Japanese SC-QALY scores are likely due to the lower Japanese cTTO scores for 64 SCRQOL states compared to those of the UK. This tendency has not been observed with some other preference-based measures; for instance, according to a EQ-5D-5L valuation study, the minimum score on EQ-5D (55555) was − 0.025 in Japan [35] and − 0.285 in the UK [36]. In the case of EQ-5D-5L, people in the UK have more negative valuations of worse health states by TTO. We cannot explain for certain why these differences arose; one possible reason is that in a Japanese ASCOT preference study, cTTO scores (normal TTO for positive score and lead time TTO for negative score) were used, whereas in the UK, normal TTO was also used for the worse-than-dead state. Minimum cTTO score is limited to –1, though normal TTO score can be less than − 1. This methodological difference might have affected the results. Another interpretation is that Japanese preferences were truly lower than UK preferences in the area of social care, but not in healthcare. If this is true, the concept of “pin pin korori” might have influenced the cTTO scores among our respondents.

Regarding the limitations of this study, respondents were not recruited using a rigid random sampling method, because the time required for the survey was assumed to be too long to do so. We judged that a door-to-door survey (i.e., where investigators visit sampled people at their residence and ask them to respond to questions at the entrance or in a room) would be difficult to conduct. The sampling method we used is similar to that described for a preference survey of EQ-5D-5L [35]. Respondents mostly had characteristics similar to the general Japanese population, although, for instance, education level was higher in our sample. This may be regarded as another limitation of our analysis from the perspective of generalizability. Second, we applied mixed logit model clustered with each respondent’s two best and two worst choices. This might imply that coefficients of the best and worst choices are heterogeneous. We assume that the absolute values of coefficients are not different between the best and worst choices; however, this may be another limitation of our analysis.

With the development of the Japanese ASCOT SCT4, it is now possible to calculate QALY for economic evaluation in the area of social care. However, some aspects of the Japanese ASCOT have not been clarified yet, because experiences with ASCOT use have not accumulated to a sufficient degree. For example, the relationship between ASCOT and other preference-based measures (e.g., EQ-5D-5L, which is the most frequently used measure for obtaining QOL scores) is unknown, although in other countries, several studies have been published [37–39]. Moreover, the population norms of SC-QALY scores may help us interpret the obtained data, for example, SC-QALY scores by care level (in Japan, long-term care insurance is provided according to 7 severity categories, with care level 5 being the most severe). A future study is warranted to address these issues. Nevertheless, the present study should serve as a catalyst to promote outcomes research in Japan, including economic evaluation in the area of social care.

### Electronic supplementary material

Below is the link to the electronic supplementary material.

Supplementary material 1 (PDF 559 kb)

## Appendix: How to calculate SC-QALY

For example, in the case that a response to ASCOT is “24313222”, the SC-QALY score can be calculated as follows:

1. Using Table 3, weights of eight item should be added. For example, the weight of level 2 in the “Control over daily life” domain is 0.954. Similarly, the weight of level 4 in the “Personal cleanliness and comfort” domain is 0.141. So, the total weight of “24313222” is 0.954 + 0.141 + 0.247 + 0.717 + 0.345 + 0.975 + 0.825 + 0.347 = 4.551.
2. Total weight calculated in 1 is converted to utility as the following equation:
  $$  {\text{SC{-}QALY}}{\mkern 1mu} {\text{score}} = (0.221 \times {\text{total}}{\mkern 1mu} {\text{weight}}) - 0.496.  $$
  In the case of “24313222”, as total weight is 4.551, SC-QALY can be calculated by (0.221 × 4.551) − 0.496 = 0.51.
